# Environmental Sustainability of Food Environments: Development and Application of a Framework in 4 cities in South Asia

**DOI:** 10.1016/j.cdnut.2024.103791

**Published:** 2024-06-11

**Authors:** Alexandra L Bellows, Anjali Ganpule, Ahmed Raza, Deksha Kapoor, Aviva Musicus, Marie L Spiker, Lindsay M Jaacks

**Affiliations:** 1 Global Academy of Agriculture and Food Systems, The University of Edinburgh, Midlothian, United Kingdom; 2 Centre for Chronic Disease Control, New Delhi, India; 3 Food and Agriculture Organization of the United Nations (FAO), Rome, Italy; 4 Department of Nutrition, Harvard T.H. Chan School of Public Health, Boston, MA, United States; 5 Department of Epidemiology, University of Washington School of Public Health, Seattle, WA, United States; 6 Food Systems, Nutrition, and Health Program, University of Washington School of Public Health, Seattle, WA, United States

**Keywords:** food environment, sustainability, food waste, food systems, framework

## Abstract

**Background:**

Food environments, where people directly engage with broader food systems, may be an important contributor to the environmental sustainability of food systems.

**Objectives:**

The primary objectives of this study were to establish a new food environment framework that considers environmental indicators and to assess data availability and gaps using data previously collected as part of a food systems survey in 4 South Asian cities.

**Methods:**

The framework was developed by conducting a structured literature review of previous food environment frameworks and in-depth interviews with content experts (*n* = 6). The framework and indicators were then mapped to data collected by consumer and vendor surveys using the Urban Food Systems Assessment Tool (UFSAT) in Ahmedabad (India), Pune (India), Kathmandu (Nepal), and Pokhara (Nepal).

**Results:**

We have expanded the sustainability domain within food environments to include consumer travel to food vendors, the presence of food delivery services, policies related to sustainability, vendor food waste, vendor plastic use, vendor utility usage, vendor recycling and waste management practices, and food packaging. Mapping the framework to existing data from 4 cities in South Asia, we found variations in food environment sustainability indicators, particularly regarding consumer transportation to food vendors, the presence of delivery services, and food waste.

**Conclusions:**

Although the majority of food environment research focuses on the availability and affordability of healthy foods, there is an urgent need to understand better how aspects of food environments contribute to environmental goals. When mapping the framework to existing food systems data, we found gaps in data on environmental sustainability in food environments and variation in indicators across settings.

## Introduction

The latest Intergovernmental Panel on Climate Change (IPCC) report concluded that total net greenhouse gas (GHG) emissions have continued to increase across all sectors, from energy to transport to agriculture, and urban areas account for an increasing share of emissions [1]. Even with the implementation of nationally determined contributions, it is likely that warming will exceed 1.5 °C during the 21st century [1]. Globally, there are large disparities in per capita GHG emissions. For example, in South Asia, GHG per capita is 2.6 tCO_2_-eq per person compared with 7.8 tCO_2_-eq/person in Europe [1]. However, the IPCC report also highlights many synergies between sustainable development and desirable outcomes for developing economies, such as reduced pollution and healthier diets [1].

With regard to diets, food systems are responsible for about one-third of GHG emissions [2]. In addition, food systems are a major source of pollution. Single-use plastic packaging, in particular, has been identified as a major global pollutant [[3], [4], [5]]. The majority of research on plastics within food systems has assessed the use of plastics in agriculture [6,7], but with the rising sale of ultraprocessed foods and take-away foods globally [8,9], more attention is needed on single-use food packaging and potential trade-offs with food safety.

To date, most research on the environmental sustainability of food systems has focused on GHG emissions associated with food consumption [10,11], and there is a need for a more diverse set of environmental sustainability indicators, particularly pollution. Other aspects of food systems, namely the food environment, have not been explored explicitly from an environmental sustainability perspective [12]. The majority of research on the food environment has focused on availability, accessibility, and affordability of food and its impacts on diet and health [[13], [14], [15]]. However, given that the food environment is where people directly engage with the broader food system [[16], [17], [18]], it represents an important point of intervention for influencing the environmental sustainability of food systems, in particular for reducing nonagriculture-related GHG emissions and pollution.

Globally, food environments are rapidly changing due to increased urbanization, economic development, and globalization [19]. Moreover, in a report released at the Conference of the Parties (COP) 26 in 2021, the Food and Agriculture Organization (FAO) concluded that in many countries, the food supply chain (off-farm), including the processing, packaging, and transport of foods, is on course to overtake agriculture (on-farm) as the largest contributor to food system-related GHG emissions [20]. In fact, it has already overtaken agriculture as the largest contributor in China, the United States, and the European Union [20]. This has important implications for climate change mitigation strategies and supports an urgent need for a framework to evaluate the environmental sustainability of food environments. In addition, measuring the sustainability of food environments will allow us to identify potential trade-offs within the sustainability domain and with other outcomes such as nutrition and health.

The primary objectives of this study were as follows: *1*) to establish a new framework that describes environmental sustainability considerations of food environments with a focus on nonagriculture-related GHG emissions and pollution and *2*) to map this framework to an existing food systems survey to describe the availability of relevant indicators within an existing tool and the variability of these indicators across different food environments in 4 South Asian cities: Ahmedabad (India), Pune (India), Kathmandu (Nepal), and Pokhara (Nepal).

## Methods

### Framework development

We conducted a structured review to assess the inclusion of sustainability in existing food environment frameworks and identify subdomains and indicators associated with sustainability within food environments. The search was conducted in February 2023 using Scopus with the search terms “food environment” AND “framework.” Results of the literature search were imported into Covidence software (Veritas Health Innovation, Melbourne, Australia) for title/abstract screening by 1 author (ALB). Peer-reviewed articles were included if they met the following criteria: *1*) included an original food environment framework, *2*) included an original definition of food environments, and/or *3*) mentioned sustainability as a component of the food environment. Articles included in this review were written in English. For eligible articles, full texts were reviewed by 1 author (ALB), and the frameworks and definitions of food environments were extracted by the same author.

Based on the findings of the structured review, a preliminary draft of the framework was developed with the goal of expanding a framework for food environments to include specific subdomains and indicators of environmental sustainability. Frameworks extracted from the structured review were used to identify subdomains and indicators in the first iteration of our expanded food environment framework. We mapped subdomains of the food environment to an adapted socioecological framework to describe components of environmental sustainability at varying levels within the food environment. Socioecological frameworks have been used previously in food systems, nutrition, and public health literature as a model to describe interconnections of a multilevel system [17,21,22]. Within food environments, we conceptualized 4 interconnected levels: the community, the vendor environment, the food vendor, and the product. Policies were represented by an arrow spanning all levels of the food environment because they can exist at each level within the food environment and influence different levels.

We then conducted in-depth interviews with 6 food and sustainability experts to collect their feedback on the framework. Experts were initially identified by authorship on a previous food environment framework (identified through the structured review) and known experts in food systems sustainability. All identified experts were invited via e-mail to participate in a virtual interview. Interviews were conducted between June 2023 and September 2023. Experts who were interviewed were asked to suggest any additional experts to interview. Each interview lasted ∼60 min. We asked interviewees for feedback on a draft of the framework, including questions regarding the novelty of the framework, addition to the literature, and comprehensiveness of the domains included. We specifically asked interviewees if there were any other aspects related to the environmental sustainability of the food environment that were missing from the current draft of the framework (Supplemental Material). All interviewees provided informed consent, and the protocol for expert interviews was reviewed and approved by the Human Ethics Research Committee at the University of Edinburgh (protocol # HERC_2023_058). The framework was iteratively updated based on feedback after each interview, and interviewees were presented with a revised version of the framework. If feedback conflicted with other interviews, 3 authors (AB, AG, LJ) discussed and made the final decision.

After finalizing the framework, we identified potential indicators for each subdomain of environmental sustainability in food environments. We prioritized indicators already included in food systems analysis tools, such as the Urban Food Systems Assessments for Nutrition (UFSAN) tool [23], described in detail further. Beyond this, several additional indicators were identified that could be collected in future market surveys or integrated as sustainability modules in existing tools.

### Mapping framework to existing food systems data

We mapped indicators proposed in the new framework to previously collected food systems data in South Asia using the UFSAN tool to identify potential gaps in existing food environment data collection tools. The UFSAN tool aims to measure the performance of food systems in providing healthy diets to constituents at the city-level [23]. Details of the tool have been published previously [23]. The tool was designed to help policymakers, development practitioners, and researchers better understand local food systems and enable data-informed decision making. The tool comprises multiple surveys for different actors in the food system, including consumers, food vendors, wholesalers, traders, and farmers. The tool prioritizes using existing methodologies to measure diets, food environments, and food supply chains. For this analysis, which focused on the food environment, we selected data from the UFSAN consumer and food vendor surveys that aligned with indicators proposed in our expanded framework on food environments. The UFSAN consumer surveys used in this study collected data on demographic characteristics, food purchasing behavior, and diet composition. The UFSAN food vendor surveys collected data on how food was transported to the vendor, types of food products sold, food storage, and food waste.

The UFSAN tool was piloted in 4 South Asian cities: Ahmedabad, India [24]; Pune, India [25]; Kathmandu, Nepal [26]; and Pokhara, Nepal [27]. Details on data collection for each city have been published previously in city-level reports [[24], [25], [26], [27]]. Briefly, consumer and food vendor surveys were conducted between October and December 2020 in 2 wards (smallest administrative level) in each city. Data were collected in the local language by trained interviewers, and all surveys were pilot-tested before implementation. In Kathmandu and Pune, consumer surveys were conducted via telephone owing to COVID-19 restrictions. In Ahmedabad and Pokhara, consumer surveys were conducted face-to-face while adhering to COVID-19 safety protocols in each city. Consumers were recruited via snowball sampling and lists of phone numbers and addresses collected from previous nutrition surveys [28]. The list of food vendors within each ward was initially derived from consumer surveys, with additional food vendors added by convenience during a walking survey of the wards. Informed consent was collected for both consumer and vendor surveys. The consumer and vendor surveys were approved by the Public Health Foundation of India Institutional Ethics Committee (TRC-IEC 444/20) and the Nepal Health Research Council (NHRC) (ERB Protocol Registration No. 713/2020 P).

For this analysis, we used data on food procurement practices (i.e., how often participants shopped at specific food vendors, distance to specific food vendors, and mode of transportation to food vendors) collected from consumer surveys. Food vendors included in the consumer survey were permanent wet markets, government ration shops, specialty stores (bakery and dairy), street vendors, mobile door-to-door vendors, grocery stores, and online food stores. In addition, we used data collected from food vendor surveys on food storage and food waste practices for 26 food items. In our analyses, we grouped items into 6 food groups: dairy, fruit and vegetables, meat and eggs, snacks, and staples. Meat and eggs were combined owing to a low number of vendors reporting to sell these products.

Descriptive statistics were used to describe variations in sustainability indicators across food groups and cities. All analyses were performed in R 4.2 [29].

## Results

### Development of framework and indicators

A total of 300 articles were identified in the Scopus search, of which 205 were excluded after the title/abstract screening. An additional 85 articles were excluded during the full-text review because they used or referred to a previously published food environment definition or framework rather than presenting an original or updated food environment definition or framework. During the full-text review stage, we identified an additional 7 articles to be included from the citations of other articles screened in the review process. We reviewed frameworks and food environment definitions from the remaining 17 articles [[16], [17], [18],[30], [31], [32], [33], [34], [35], [36], [37], [38], [39], [40], [41], [42], [43]]. Only 2 articles explicitly mentioned environmental sustainability as a component of the food environment [17,30]. In their framework, Downs et al. [17] defined sustainability as “the environmental and social impact associated with the food item.” Mikkelsen et al. [30] referred to sustainable foodscapes, which encompass the way “food is produced, purchased or obtained, prepared, and consumed,” but did not define indicators of sustainability within foodscapes (a term related to food environments) [30]. No definition included indicators of sustainability beyond product properties.

**FIGURE 1:**
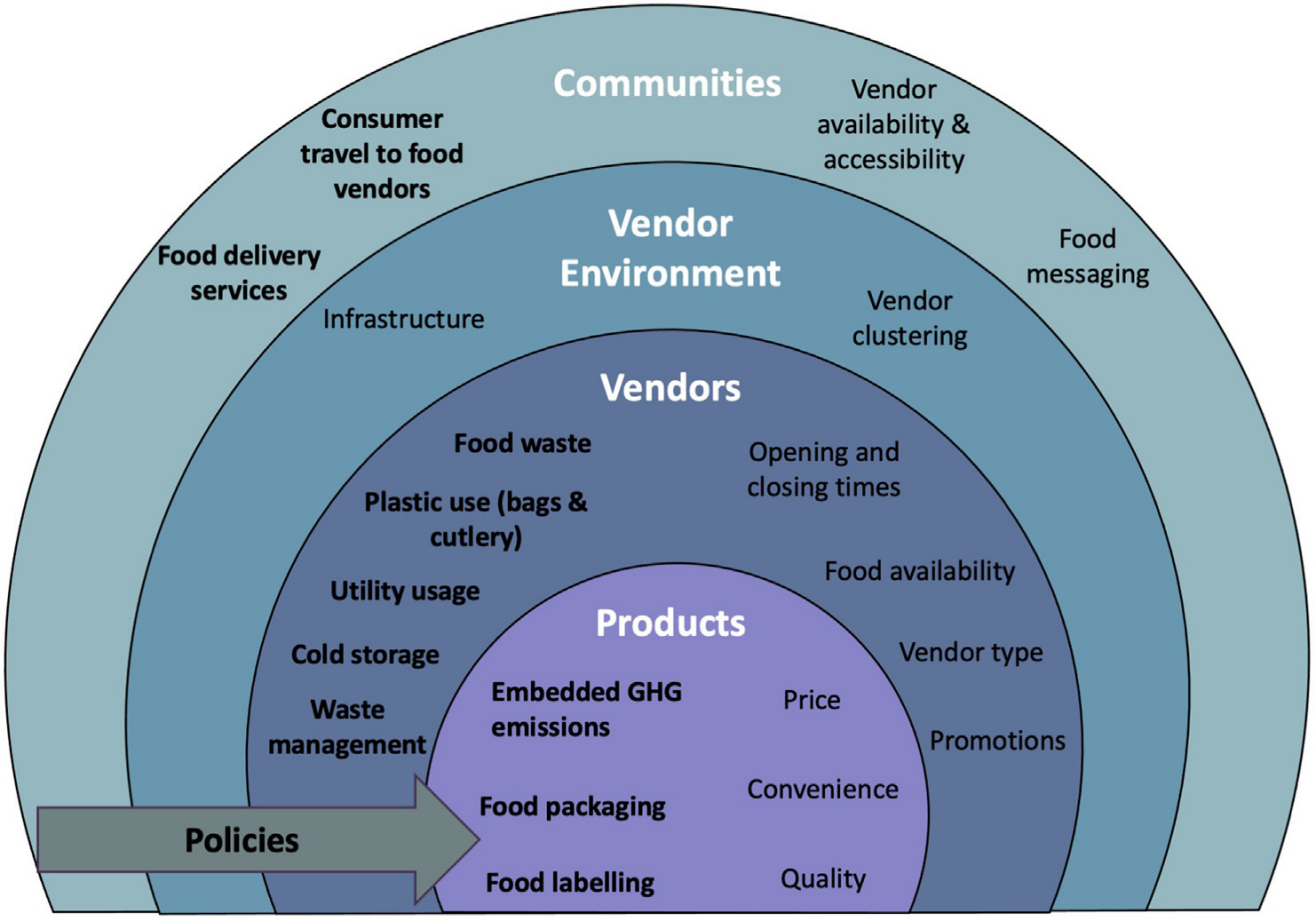
**Conceptual framework of environmental sustainability in food environments.** Subdomains highlighted in bold represent environmental components of the food environment that have not been included in previous food environment frameworks. GHG, greenhouse gas.

We proposed 12 additional subdomains within food environments: consumer travel to food vendors, the presence of food delivery services, policies related to sustainability, vendor food waste, vendor plastic use, vendor utility usage, vendor recycling and waste management practices, and food packaging (Figure 1). The majority of these subdomains have not been included in previous food environment frameworks, according to the results of the structured review. Subdomains related to environmental sustainability focused on GHG emissions and pollution as those were considered the most relevant to the food environment by experts interviewed. Other subdomains within the food environment framework were included based on data extracted from the literature structured review (Supplemental Table 1) and feedback from qualitative expert interviews. In addition, we proposed 22 food environment indicators related to GHG emissions or pollution (Table 1). Of these 22 indicators, 8 were present in the UFSAN tool, covering the subdomains of consumer travel, food service delivery, food waste, cold storage, and the environmental impact of food available.

**TABLE 1:** List of indicators to measure environmental sustainability of food environments

Category	Component	Potential indicators	Greenhouse gas emissions	Environmental degradation	Available in UFSAN tool
Community properties	Consumer travel to food vendors	What form of transportation does the consumer use to travel to food vendors?	x	x	Yes
	Food delivery services	Presence of food delivery service		x	Yes
		Type of food delivery service transportation	x	x	No
	Policies	Is there a policy or incentive for food businesses to reduce truck movements in the city?	x	x	No
		Is there a policy or incentive for food businesses to invest in equipment or technologies to avoid pollution?		x	No
		Is there a policy or plan to reduce food loss and waste in the urban food system?	x		No
		Is there a policy or plan to promote recycling of organic waste and food packaging materials and use of food byproducts?	x	x	No
Vendor properties	Food waste	How much food does the vendor not sell?	x		Yes
		What does the vendor do with food not sold?	x		Yes
		What strategies is the vendor implementing to reduce food waste?	x		Yes
	Plastic use (bags and cutlery)	Does food vendor provide plastic bags? Does food vendor provide plastic cutlery?		x	No
	Utility usage	Amount of electricity and water used by vendor	x	x	No
		Does the vendor have electricity?	x		No
		Does the vendor have air-conditioning?	x		No
	Cold storage	Where does the vendor store most food?			Yes
		Does the vendor store food items in refrigerators?	x		Yes
	Waste management	Does the vendor have a recycling system in place?		x	No
		Does the vendor have a waste management system in place?		x	No
Product properties	Environmental impact	Availability of animal-source foods	x		Yes
	Food packaging	Type of packaging used for food available: plastic, paper, none, other		x	No
	Food labeling	Does product have an ecolabel? If yes, what type?	x	x	No

### Mapping framework to existing food systems data

Although detailed city-level results of the consumer and food vendor surveys are published elsewhere, in this study, we highlight consumer and vendor characteristics that are related to the environmental sustainability of food environments. A total of 1797 consumers were surveyed across the 4 cities: Ahmedabad (*n* = 446), Pune (*n* = 451), Kathmandu (*n* = 450), and Pokhara (*n* = 450) (Supplemental Table 2). In Kathmandu and Pokhara, 55 food vendors were surveyed in each city. In Ahmedabad and Pune, 54 and 66 food vendors were surveyed, respectively (Supplemental Table 3). Across all cities, consumers reported a high reliance on wet markets and small local shops for weekly shopping (Supplemental Table 4). For dairy, there was also a high reliance on specialty stores in all cities (Supplemental Table 5).

**FIGURE 2:**
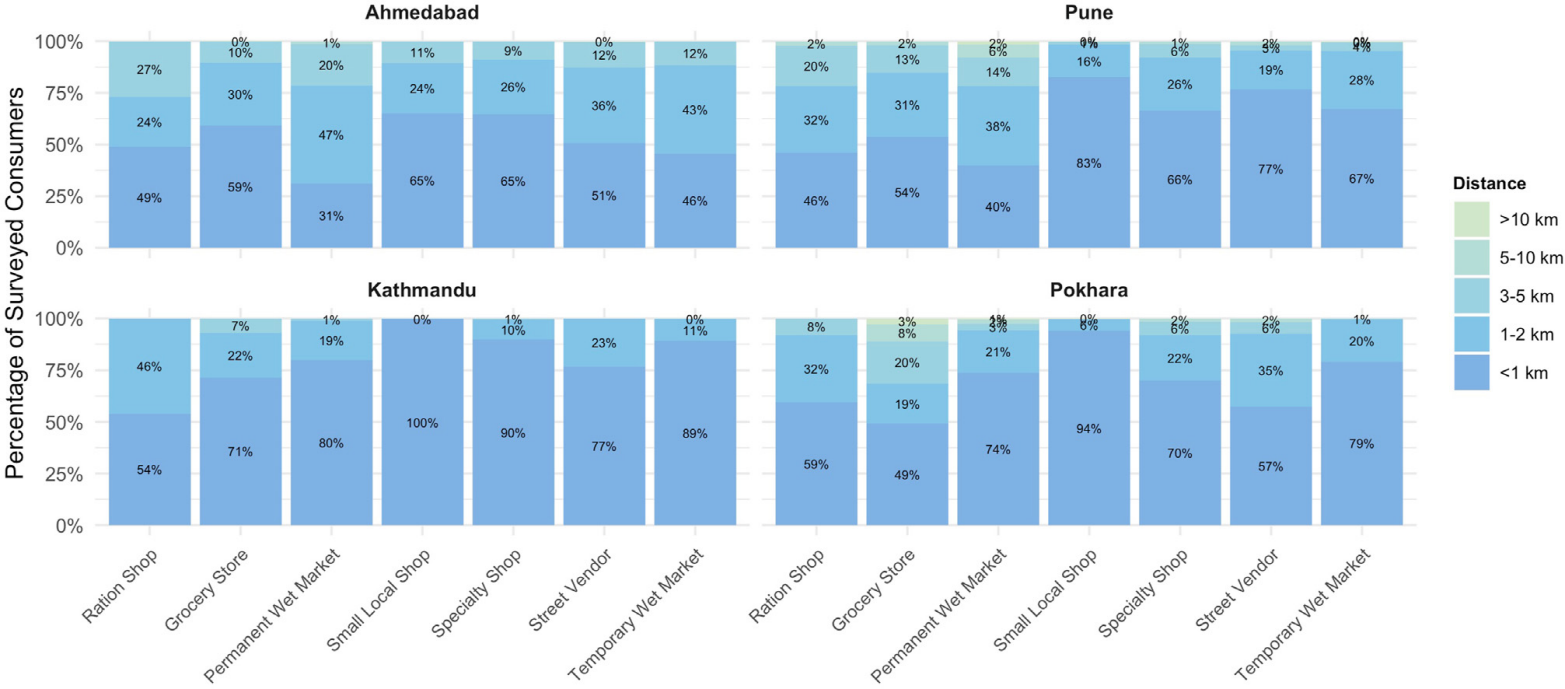
**Distance to the nearest food vendor by food vendor type for consumer respondents.** Total number of respondents for each city: Ahmedabad (*n* = 446), Pune (*n* = 451), Kathmandu (*n* = 450), and Pokhara (*n* = 450). In consumer surveys, participants were asked if they shopped at each food vendor type. If yes, the participants were then asked to report the approximate distance to the nearest vendor. The total number of respondents for each vendor type by city is reported in Supplemental Table 7.

For nearly all food vendors across the 4 cities, most consumers reported walking as their primary form of transportation. The only exceptions were government ration shops and permanent wet markets in Ahmedabad and Pune, where motorbike was the main form of transportation reported (Supplemental Table 6). The percentage of consumers reporting using cars, motorbikes, or public transport was very low in Kathmandu and Pokhara, with <10% reporting using motorbikes and <1% reporting driving a personal vehicle. In contrast, in both Ahmedabad and Pune, the majority of participants, 64% and 59%, respectively, reported motorbike was their main form of transport to ≥1vendor. The use of personal vehicles was highest in Pune, where 15% of consumers reported car use as the main form of transportation to ≥1 type of vendor (Table 2). This contrast between the cities in Nepal and India could be related to the distance to the nearest vendor; vendors tended to be further away in India (Figure 2).

**TABLE 2:** Percentage of surveyed consumers reporting regular use of ≥1 nonwalking or nonbicycle ravel mode in each city

Transport	Ahmedabad	Pune	Kathmandu	Pokhara
Car	0.4 (2)	15.5 (70)	0.4 (2)	0.4 (2)
Motorbike	63.7 (284)	59.2 (267)	9.8 (44)	9.8 (44)
Public transport	7.2 (32)	6.0 (27)	2.4 (11)	8.2 (37)

All values are reported as % (*n*).

Food delivery services were available in all 4 cities, with the majority of food delivery services being mobile door-to-door vendors (Supplemental Table 4). Nearly two-thirds of consumers in Ahmedabad, Kathmandu, and Pokhara reported purchasing food from a mobile door-to-door vendor in the last month, compared with 20% in Pune (Supplemental Table 7). About a third of surveyed consumers in India reported using online food vendors to purchase food in the last month, in contrast to only 3% of surveyed consumers in Nepal (Supplemental Table 7). In all cities, few consumers reported using online food vendors to procure food more than once per week (Supplemental Table 4). No information was available within the UFSAN data set on the mode of transport for the delivery of food from mobile and online vendors.

Across all 4 cities, few vendors surveyed reported selling meat, which typically is associated with the highest GHG emissions. For other animal-source foods, a slightly higher proportion of vendors surveyed in Kathmandu and Pokhara reported selling dairy, and a significantly higher proportion of surveyed vendors reported selling eggs than in Ahmedabad and Pune (Table 3).

**TABLE 3:** Percentage of surveyed food vendors reporting selling each food group

Food group	Ahmedabad (*n* = 54)	Pune (*n* = 66)	Kathmandu (*n* = 55)	Pokhara (*n* = 55)
Dairy	13.0 (7)	24.2 (16)	38.2 (21)	30.9 (17)
Eggs	5.5 (3)	7.5 (5)	41.8 (23)	49.1 (27)
Fruit and vegetables	23.6 (13)	18.2 (12)	30.9 (17)	38.2 (21)
Meat	3.6 (2)	1.5 (1)	3.6 (2)	9.1 (5)
Snacks	36.4 (20)	66.7 (44)	78.2 (43)	78.2 (43)
Staples	16.7 (9)	27.3 (18)	47.3 (26)	50.9 (28)

All values are reported as % (*n*). Data are from a convenience sample of food vendors and should not be considered representative of each city.

**FIGURE 3:**
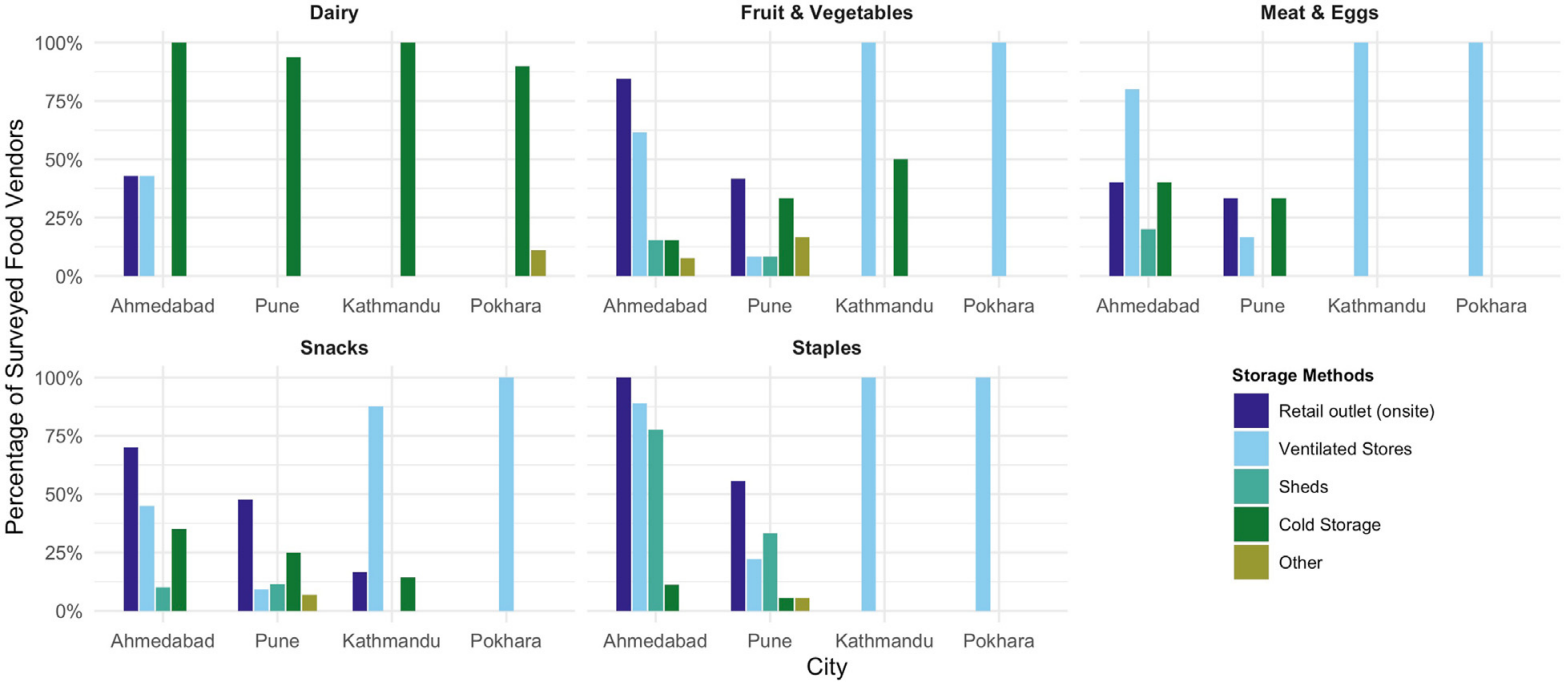
**Food vendors’ reported storage method by food group.** Total number of food vendors in each city: Ahmedabad (*n* = 54), Pune (*n* = 66), Kathmandu (*n* = 55), and Pokhara (*n* = 55). For each food item stored, food vendors reported any food storage method used. Food vendors were able to select >1 strategy. Therefore, values may exceed 100%.

Cold storage was available for vendors surveyed across all 4 cities. The majority of vendors reported cold storage usage for dairy products (Figure 3). For fruits, vegetables, snacks, and staple grains, vendors reported storing most food in ventilated storage units (units that contain air vents or openings to prevent food waste) or at the retail site. In Kathmandu, 50% of vendors selling fruits and vegetables reported using cold storage to store ≥1 fruit or vegetable.

**FIGURE 4:**
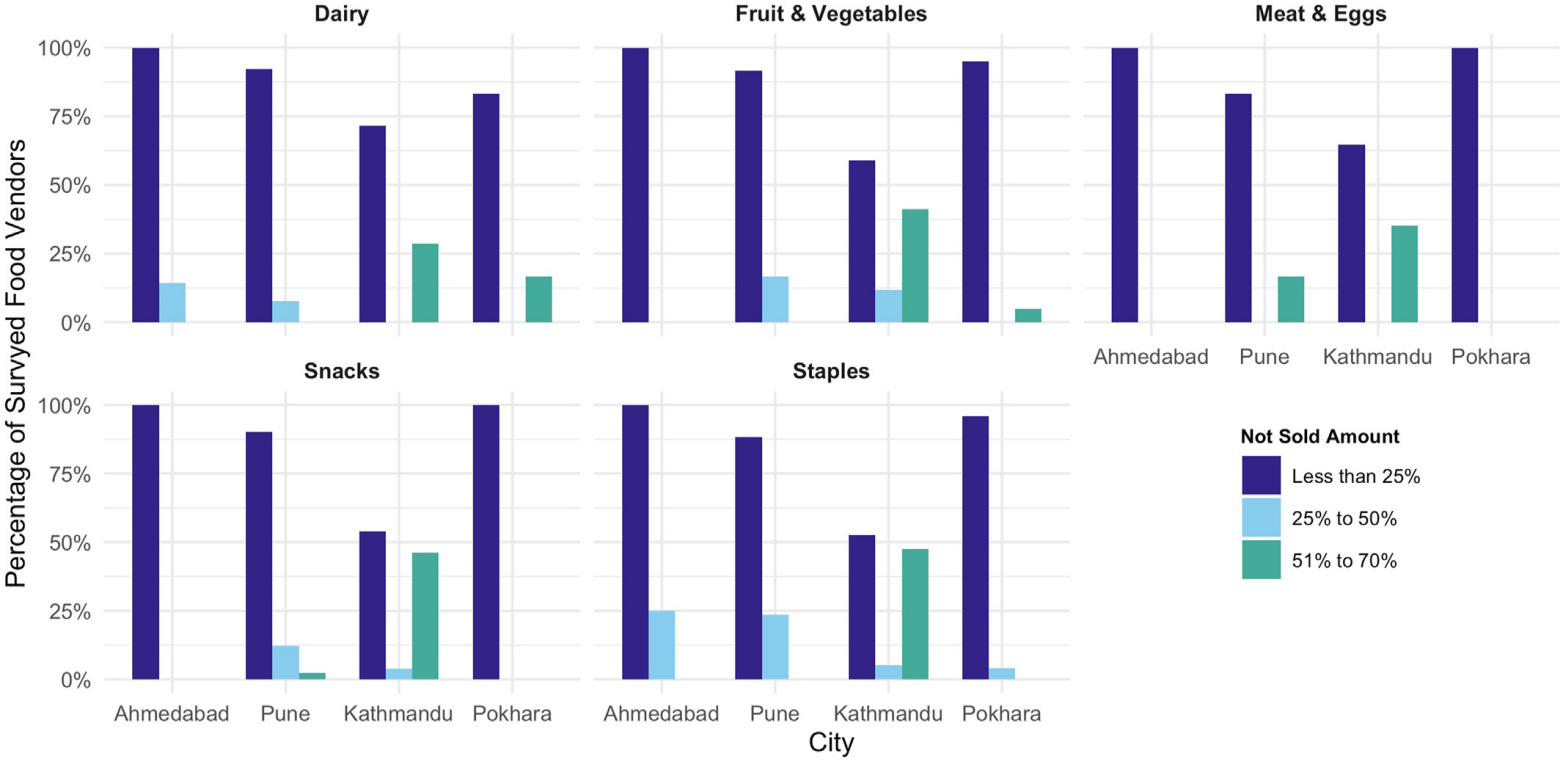
**Food vendors’ reported proportion of food not sold by food group.** Total number of food vendors in each city: Ahmedabad (*n* = 54), Pune (*n* = 66), Kathmandu (*n* = 55), and Pokhara (*n* = 55). For each food item sold, vendors reported any percentage of food not sold by selecting 1 of 3 categories: <25%, 25%–50%, and 51%–70%. Because items were aggregated to food group level, values may exceed 100%.

**FIGURE 5:**
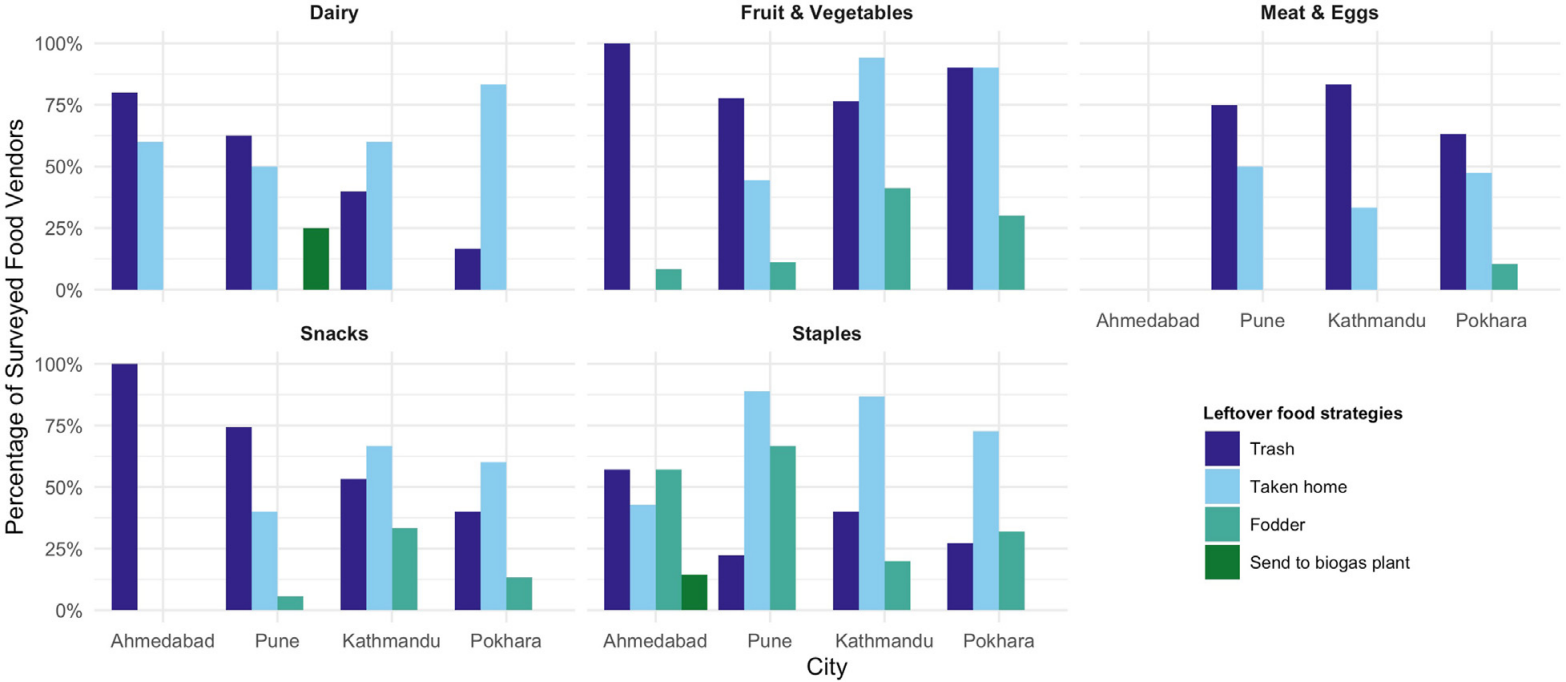
**What happens to leftover food at food vendors by food group?** Total number of food vendors surveyed in each city: Ahmedabad (*n* = 54), Pune (*n* = 66), Kathmandu (*n* = 55), and Pokhara (*n* = 55). For each food item sold, vendors reported what happens to items not sold. Retailers were able to select >1 strategy. Therefore, values may exceed 100%. There is no data reported for vendors selling meat or eggs in Ahmedabad because all vendors responded that there were no leftover food items.

**FIGURE 6:**
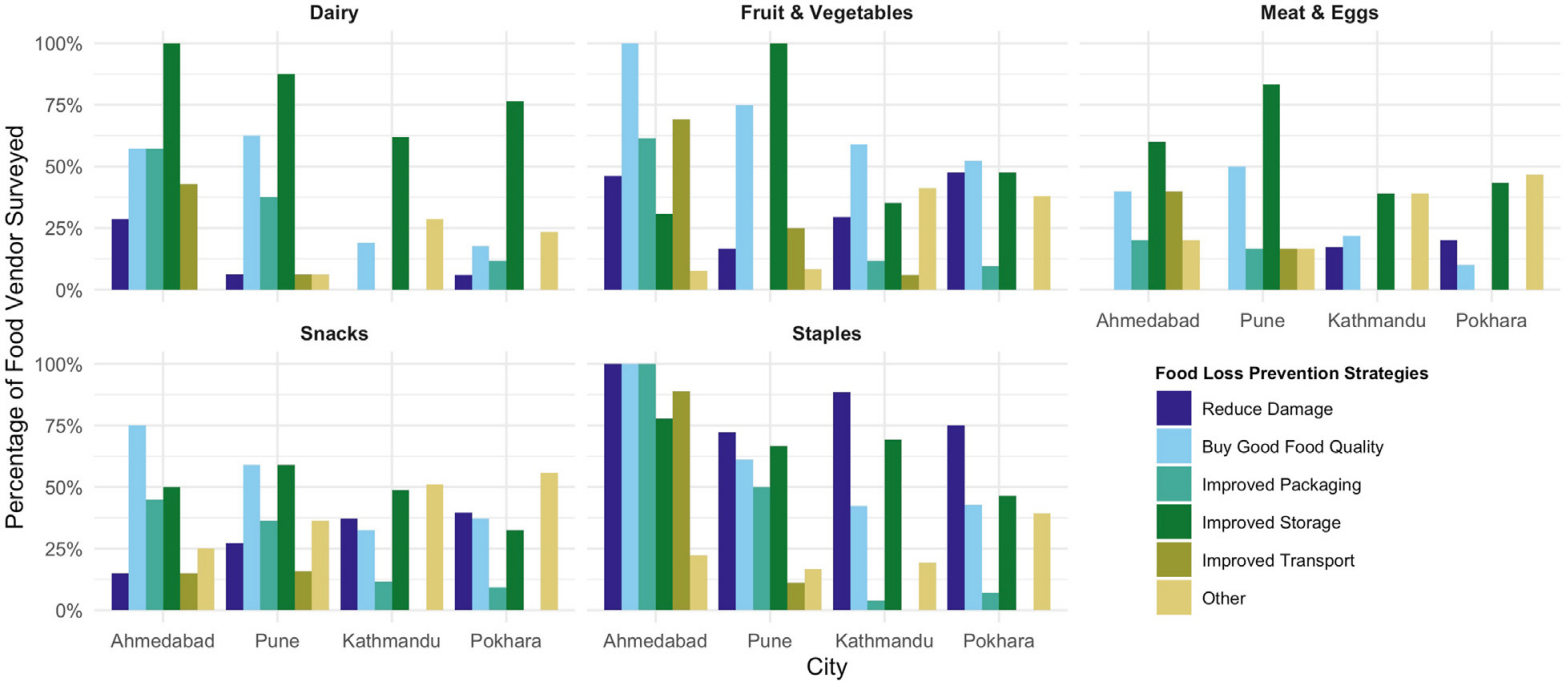
**Strategies reported by food vendors to reduce food loss**. Total number of food vendors in each city: Ahmedabad (*n* = 54), Pune (*n* = 66), Kathmandu (*n* = 55), and Pokhara (*n* = 55). For each food item sold, vendors reported any food loss prevention strategies used. Retailers were able to select >1 strategy. Therefore, values may exceed 100%.

Most vendors reported that <25% of food products available in their establishments were not sold (Figure 4). In Kathmandu, >25% of vendors reported high food waste (51%–70% of products not sold) for dairy, eggs, fruits and vegetables, snacks, and staple products. For food not sold, vendors reported multiple methods for food disposal. The majority of vendors reported that most food across food groups was either disposed of in the trash or taken home by employees. Meat and eggs were more likely to be disposed of in the trash, while staples were more likely to be taken home by employees or sent to fodder (Figure 5). Variations in strategies for dealing with food not sold were seen across cities, with vendors in Nepal slightly more likely to encourage food not sold to be taken home by employees. Vendors also reported a variety of strategies to prevent food waste (Figure 6). Improved storage was highlighted as a key initiative across all cities for dairy, eggs, and meat. For fruits and vegetables, buying good quality food was another strategy that all vendors across the 4 cities reported. For staples, reducing damage through protective activities such as cleaning and disinfecting storage containers was a major strategy highlighted by vendors across all 4 cities.

## Discussion

This study aimed to advance food systems research by proposing a set of indicators to measure the environmental sustainability of food environments and identify gaps in current food environment tools using existing food environment data from 4 South Asian cities. Identifying indicators that can be incorporated into existing food environment surveys (such as consumer and market-based surveys) will allow for the ready adoption of these measures into food environment analyses. In addition, once data has been collected, these indicators can be linked to local life-cycle assessment and pollution estimates to quantify the environmental impact of food environments.

Before this study, only 2 other articles incorporated sustainability into their definitions of food environments [17,30]. Downs et al. [17] proposed 6 domains within the food environment: availability, affordability, promotion, convenience, quality, and sustainability. Within the sustainability domain, both studies focused on the sustainability of the food items available—for example, the GHG emissions embedded in the production of those food items. Through a structured review of previous food environment frameworks and definitions and through expert interviews, we propose a set of considerations that expand our understanding of the environmental sustainability domain of food environments: consumer travel to food vendors, the presence of food delivery services, policies related to sustainability, vendor food waste, vendor plastic use, vendor utility usage, vendor recycling and waste management practices, and food packaging. Current food environment assessments can be expanded to collect data on these additional elements of food environment sustainability.

Mapping the framework to existing data from 4 cities in South Asia, we found that the majority of indicators for measuring environmental sustainability were not available. This highlights an urgent need for tools to better incorporate aspects of environmental sustainability into their food environment assessments. For environmental indicators that were present in the existing data, we found variations in food environment sustainability, particularly regarding consumer transportation to food vendors. Pune had the highest car usage, with 15% of respondents reporting traveling by car to purchase food and the greatest portion of vendors >1 km from consumer homes. When comparing these numbers with those of other urban areas, consumer travel within these urban food environments in South Asia is more environmentally sustainable and in line with some recommendations for a 15-min neighborhood—neighborhoods in which people can access their daily needs within a 15-min walk or cycle [44,45]. For example, a study in Seattle found that 88% of respondents used a motorized vehicle to travel to the grocery store and that the distance to their primary grocery store was, on average, 4 km from their home [46]. In this study, both car ownership and distance to primary grocery store were main predictors for driving to the grocery store. Although Seattle may not be directly comparable with cities such as Pune and Kathmandu, it is important to highlight that the proximity to food vendors within these contexts may be promoting more sustainable food procurement methods compared with that in some cities in North America and Europe.

In addition, the availability and use of online food vendors are continuing to grow both in South Asia and globally. The presence of online food vendors has important implications for both sustainability and nutrition outcomes and needs to be better incorporated into food environment assessments [47]. In particular, we need more information on typical modes of transportation online and mobile vendors use to deliver food to understand potential impacts on sustainability and the types of food typically delivered using these services.

At the food vendor level, the majority of food vendors reported selling 75% or more of their products. Food vendors selling more perishable food items such as dairy, meat, eggs, fruits, and vegetables did not appear to report higher amounts of food waste compared to food vendors selling snacks and staples. This could be because most food loss/waste in these contexts likely occurs at the farm level or during storage and handling before reaching the food vendor. A report estimating food loss and waste across the food system estimated that nearly 70% of food loss occurred at the production (30%) or handling and storage (37%) stages of the food supply chain in South and Southeast Asia [48]. Although consumer underreporting of household food waste has been noted in high-income country contexts (the United States and United Kingdom) [49,50], the extent of potential underreporting among vendors in this context is not known, particularly because the majority of food waste data for food services and retail are from studies conducted in the United States and Western Europe [51]. It should also be noted that South and Southeast Asia accounted for 17% of food lost or wasted globally, while North America and Oceania accounted for 42% of food lost or wasted [48]. In addition, for food items that are considered more valuable, there may be increased efforts to reduce waste. For example, globally, meat and dairy comprise a relatively low portion of losses worldwide despite being highly perishable [48]. Few food vendors reported seeking improved transport methods or packaging as food waste prevention strategies. This may be because these strategies are beyond the food vendor’s control, and decision-makers should target more upstream food system stakeholders.

The availability of animal-source foods, with the exception of dairy, was low in vendors surveyed for all 4 South Asian cities. This may reflect the dietary patterns of consumers in these cities. For example, one of the wards in Ahmedabad city has a high prevalence of Jain population who abstain from meat and eggs. In Ahmedabad, 61.5% of consumers surveyed reported 4-6 individuals in their household followed a strictly vegetarian diet (no consumption of meat or eggs) [24].

Potential trade-offs exist within the sustainability domain. For example, cold chains may increase GHG emissions and pollution because of high electricity demand, fossil fuel usage, and refrigerant usage, but they can also reduce losses and increase food safety [52]. Globally, cold chains are responsible for 4% of GHG emissions [53], and refrigerant management has been identified as a key climate initiative by Project Drawdown [54]. On the contrary, ∼12% of total food production is lost owing to inadequate refrigeration [53] and with global warming, this estimate is likely to increase. In addition, researchers have identified improving and increasing access to cold chain storage as a key policy to promote healthier and safer diets [55]. Foods with high nutrient density, such as fruits, vegetables, and animal-source foods, are typically more perishable than staples and packaged snack foods [56]. Therefore, when assessing the sustainability of food environments, a food group approach should be considered to avoid rewarding food vendors with minimal food waste and limited cold storage but only selling highly processed packaged foods. Collecting data on the cold chain storage type would be useful for identifying food vendors and food environments that promote innovative cold storage solutions.

Another trade-off within the sustainability domain exists between packaging and food waste. Packaging can help improve food safety and food waste, especially packaging innovations that are optimized for waste reduction; however, some packaging—plastic, in particular—contributes to both GHG emissions and pollution and can have food safety concerns related to microplastics [5]. Plastic production requires the extraction of natural resources, resulting in GHG emissions, and globally, it is estimated that 42% of plastic packaging will end up in unregulated dumpsites, burned, or trash, rather than being recycled [57]. To reduce plastic waste, many countries, including India [58], have implemented plastic bag bans, but the use of plastic in most food systems extends far beyond the use of plastic bags. Plastic food packaging has been associated with increased exposure to bisphenols and phthalates, both known endocrine disruptors [[59], [60], [61]]. On the contrary, there has been increased interest in biodegradable packaging as a solution to reduce plastic in the food system, but more research is needed to identify potential trade-offs between food waste, pollution, and food safety [62]. Identifying the type of packaging used for certain food items and differences in packaging types across food environments can help illuminate where policies to promote innovative packaging solutions would be most effective.

Moreover, packaging allows for the presence of nutrition and ecolabels, which may help consumers make healthier and more sustainable choices. For example, policies to promote front-of-pack warning signs on foods have been adopted in a few countries to combat the rising prevalences of overweight and obesity [63]. More recently, food companies have begun adding ecolabels to products, and a systematic review has shown that these labels can promote the purchasing of more sustainable products [64].

This analysis leveraged multiple methods—literature search, expert interviews, and previously collected food systems data—to develop a novel framework and identify future research needs to advance research on the environmental sustainability of food environments. Limitations of this analysis include the reliance on secondary data that were collected before the development of the framework, which resulted in missing data for many environmental sustainability subdomains. Further development of a market-based survey is necessary to collect all aspects of environmental sustainability. In addition, for the UFSAN data, we relied on pilot data for each city and both consumer and food vendor surveys relied on a convenience sample. Consumer data for the UFSAN pilot were collected using snowball sampling, and all data were collected during the COVID-19 pandemic, which may have impacted food consumed by residents, modes of transportation for consumers, and food available at vendors. Therefore, the results are not generalizable to each city. The purpose of the UFSAN data was to highlight the availability of data related to food environment sustainability and show how environmental sustainability subdomains and indicators may vary within different food environments.

In conclusion, food systems need to undergo a rapid transformation to provide sustainable and healthy diets. Although the majority of food environment research focuses on the availability and affordability of healthy foods, we argue that there is also an urgent need to understand better how aspects of food environments contribute to our environmental goals. Sustainability research within food systems has primarily focused on which foods people are choosing and the embedded resource use and impacts of those foods. This work helps to broaden the points of intervention for sustainable food systems to also include food environments.

## Acknowledgments

We thank Dr Prabhakaran Dorairaj and Dr Manu Mathur at the Public Health Foundation of India for their support in survey development and data collection; the teams of data collectors in each city: Himanshi Pandey and Shireen Lobo from PHFI, Delhi; Bhushana Karandikar and team from Pune; Ashok Jadeja and team from Ahmedabad; and Nira Joshi and team from Nepal; and Dr Shauna Downs for her input on the framework and feedback on the initial draft of the manuscript.

### Authors contributions

The authors’ responsibilities were as follows – ALB, AG, LMJ: designed the research project; ALB, DK: conducted expert interviews; ALB: analyzed data and performed statistical analysis; MS, AM: gave extensive feedback on framework, indicators, and manuscript; ALB: wrote the initial draft article and had primary responsibility for final content; and all authors: read and approved the final manuscript.

## Conflicts of interest

The authors report no conflicts of interest or personal relationships that could have appeared to influence the work reported in this article. AM is also affiliated with the Center for Science in the Public Interest but contributed to this article entirely under her affiliation with Harvard.

## Funding

AB was supported by IMMANA Fellowship (2022-2023), funded jointly by the UK Foreign Commonwealth and Development OFfice as grant number 300654 and the Bill and Melinda Gates Foundation as INV-002962. The Innovative Methods and Metrics for Agriculture and Nutrition Actions (IMMANA) programme is managed by the London School of Hygiene and Tropical Medicine (LHSTM) and the IMMANA Fellowship worksteam is managed by Tufts University. AG and LMJ were supported by IMMANA mentorship. AB and LMJ were supported by the Medical Research Council/UK Research and Innovation (grant number): MR/T044527/1. AM was supported by grants T32HL098048 and T32CA057711 from the NIH.

## Data availability

Data described in the manuscript, code book, and analytic code will be made publicly and freely available without restriction at www.github.com/albellows/UFSAN_Sustain_FE.

## Appendix A Supplementary data

Multimedia component1 Multimedia component1

Multimedia component2 Multimedia component2

## References

[1] Pörtner HO, Roberts D.C., Tignor M., Poloczanska E., Mintenbeck K., Alegría A. (2022). Climate change 2022: Impacts, Adaptation, and Vulnerability Contribution of Working Group II to the Sixth Assessment Report of the Intergovernmental Panel on Climate Change.

[2] Crippa M., Solazzo E., Guizzardi D., Monforti-Ferrario F., Tubiello F.N., Leip A. (2021). Food systems are responsible for a third of global anthropogenic GHG emissions. Nat. Food..

[3] United Nationals Environment Programme (2021).

[4] Yates J., Gillespie S., Savona N., Deeney M., Kadiyala S. (2021). Trust and responsibility in food systems transformation. Engaging with Big Food: marriage or mirage?. BMJ Glob Health.

[5] FAO (December 2021). http://www.fao.org/documents/card/en/c/cb7856en.

[6] Yates J., Deeney M., Rolker H.B., White H., Kalamatianou S., Kadiyala S. (2021). A systematic scoping review of environmental, food security and health impacts of food system plastics. Nat. Food..

[7] Yates J., Deeney M., Rolker H.B., White H., Kalamatianou S., Kadiyala S. (2021). Effects of plastics in the food system on human health, food security, and the environment: a systematic scoping review. Lancet Planet Health.

[8] Baker P., Machado P., Santos T., Sievert K., Backholer K., Hadjikakou M. (2020). Ultra-processed foods and the nutrition transition: global, regional and national trends, food systems transformations and political economy drivers. Obes. Rev..

[9] Vandevijvere S., Jaacks L.M., Monteiro C.A., Moubarac J.C., Girling-Butcher M., Lee A.C. (2019). Global trends in ultraprocessed food and drink product sales and their association with adult body mass index trajectories. Obes. Rev..

[10] Willett W., Rockström J., Loken B., Springmann M., Lang T., Vermeulen S. (2019). Food in the Anthropocene: the EAT–Lancet Commission on healthy diets from sustainable food systems. Lancet.

[11] Green R.F., Joy E.J.M., Harris F., Agrawal S., Aleksandrowicz L., Hillier J. (2018). Greenhouse gas emissions and water footprints of typical dietary patterns in India. Sci Total Environ.

[12] Fanzo J., Bellows A.L., Spiker M.L., Thorne-Lyman A.L., Bloem M.W. (2021). The importance of food systems and the environment for nutrition. Am. J. Clin. Nutr..

[13] Turner C., Kalamatianou S., Drewnowski A., Kulkarni B., Kinra S., Kadiyala S. (2020). Food environment research in low- and middle-income countries: a systematic scoping review. Adv. Nutr..

[14] Caspi C.E., Sorensen G., Subramanian S.V., Kawachi I. (2012). The local food environment and diet: a systematic review. Health Place.

[15] Lytle L.A., Sokol R.L. (2017). Measures of the food environment: a systematic review of the field, 2007–2015. Health Place.

[16] Turner C., Aggarwal A., Walls H., Herforth A., Drewnowski A., Coates J. (2018). Concepts and critical perspectives for food environment research: a global framework with implications for action in low- and middle-income countries. Glob Food Secur.

[17] Downs S.M., Ahmed S., Fanzo J., Herforth A. (2020). Food environment typology: advancing an expanded definition, framework, and methodological approach for improved characterization of wild, cultivated, and built food environments toward sustainable diets. Foods.

[18] Herforth A., Ahmed S. (2015). The food environment, its effects on dietary consumption, and potential for measurement within agriculture-nutrition interventions. Food Secur.

[19] Downs S.M., Ahmed S., Warne T., Fanzo J., Loucks K. (2022). The global food environment transition based on the socio-demographic index. Glob Food Secur.

[20] Tubiello F.N., Karl K., Flammini A., Gütschow J., Obli-Layrea G., Conchedda G. (2021). Pre- and post-production processes along supply chains increasingly dominate GHG emissions from agri-food systems globally and in most countries. Earth Syst. Sci. Data Discuss.

[21] Food Systems Dashboard (2024). https://www.foodsystemsdashboard.org/information/about-food-systems#a-food-systems-framework.

[22] McLeroy K.R., Bibeau D., Steckler A., Glanz K. (1988). An ecological perspective on health promotion programs. Health Educ. Q..

[23] Raza A., Jaacks L.M., Ganpule-Rao A.V., Pandey H., Lobo A. (2022). http://www.fao.org/documents/card/en/c/cb8612en.

[24] Raza A., Pandey H., Lobo A.S., Ganpule-Rao A.V. (2022). http://www.fao.org/documents/card/en/c/cb8621en.

[25] Raza A., Pandey H., Lobo A.S., Ganpule-Rao A.V. (2022).

[26] Raza A., Pandey H., Lobo A.S., Ganpule-Rao A.V. (2022). http://www.fao.org/documents/card/en/c/cb8630en.

[27] Raza A., Pandey H., Lobo A.S., Ganpule-Rao A.V. (2022). http://www.fao.org/documents/card/en/c/cb8620en.

[28] Home, Pune Municipal Corporation BINDI—The Birmingham-India Nutrition Initiative. https://www.pmc.gov.in/en/Birmingham-India-nutrition.

[29] R Core Team, R (2022). https://www.R-project.org/.

[30] Mikkelsen B.E. (2011). Images of foodscapes: introduction to foodscape studies and their application in the study of healthy eating out-of-home environments. Perspect. Public Health.

[31] Glanz K., Sallis J.F., Saelens B.E., Frank L.D. (2007). Nutrition Environment Measures Survey in Stores (NEMS-S): development and evaluation. Am. J. Prev. Med..

[32] Green S.H., Glanz K. (2015). Development of the perceived nutrition environment measures survey. Am. J. Prev. Med..

[33] Swinburn B., Vandevijvere S., Kraak V., Sacks G., Snowdon W., Hawkes C. (2013). Monitoring and benchmarking government policies and actions to improve the healthiness of food environments: a proposed Government Healthy Food Environment Policy Index: monitoring public sector policies and actions. Obes. Rev..

[34] Booth A., Barnes A., Laar A., Akparibo R., Graham F., Bash K. (2021). Policy action within urban African food systems to promote healthy food consumption: a realist synthesis in Ghana and Kenya. Int. J. Health Policy Manag.

[35] Mayer K. (2009). Childhood obesity prevention: focusing on the community food environment. Fam. Commun. Health..

[36] Cong N., Zhao A., Kwan M.P., Yang J., Gong P. (2022). An indicator measuring the influence of the online public food environment: an analytical framework and case study. Front. Nutr..

[37] Raza A., Fox E.L., Morris S.S., Kupka R., Timmer A., Dalmiya N. (2020). Conceptual framework of food systems for children and adolescents. Glob Food Secur.

[38] Murphy M., Badland H., Koohsari M.J., Astell-Burt T., Trapp G., Villanueva K. (2017). Indicators of a health-promoting local food environment: a conceptual framework to inform urban planning policy and practice. Health Promot J Aust.

[39] Clary C., Matthews S.A., Kestens Y. (2017). Between exposure, access and use: reconsidering foodscape influences on dietary behaviours. Health Place.

[40] Bogard J.R., Andrew N.L., Farrell P., Herrero M., Sharp M.K., Tutuo J. (2021). A typology of food environments in the pacific region and their relationship to diet quality in Solomon Islands. Foods.

[41] Toure D., Herforth A., Pelto G.H., Neufeld L.M., Mbuya M.N.N. (2021). An emergent framework of the market food environment in low- and middle-income countries. Curr. Dev. Nutr..

[42] Sawyer A.D.M., van Lenthe F., Kamphuis C.B.M., Terragni L., Roos G., Poelman M.P. (2021). Dynamics of the complex food environment underlying dietary intake in low-income groups: a systems map of associations extracted from a systematic umbrella literature review. Int. J. Behav. Nutr. Phys. Act..

[43] Franco M., Bilal U., Díez J. (2015). Food environment. Encyclopedia of Food and Health.

[44] Allam Z., Nieuwenhuijsen M., Chabaud D., Moreno C. (2022). The 15-minute city offers a new framework for sustainability, liveability, and health. Lancet Planet Health.

[45] Moreno C., Allam Z., Chabaud D., Gall C., Pratlong F. (2021). Introducing the “15-minute city”: sustainability, resilience and place identity in future post-pandemic cities. Smart Cities.

[46] Jiao J., Moudon A.V., Drewnowski A. (2011). Grocery shopping how individuals and built environments influence choice of travel mode. Transp. Res. Rec..

[47] Granheim S.I., Løvhaug A.L., Terragni L., Torheim L.E., Thurston M. (2022). Mapping the digital food environment: a systematic scoping review. Obes. Rev..

[48] Lipinski B., Hanson C., Lomax J., Kitinoja L., Waite R., Searchinger T. (2013). Creating a sustainable food future, installment two.

[49] Neff R.A., Spiker M.L., Truant P.L. (2015). Wasted food: U.S. consumers’ reported awareness, attitudes, and behaviors. PLoS One.

[50] Quested T., Ingle R., Parry A. (2013).

[51] United Nations Environmental Programme (2021).

[52] Trotter P.A., Becker T., Renaldi R., Wang X., Khosla R., Walther G. (2023). The role of supply chains for the sustainability transformation of global food systems: a large-scale, systematic review of food cold chains. J Ind. Ecol..

[53] UNEP; FAO (2022). http://www.fao.org/documents/card/en/c/cc0923en.

[54] (2020). Project Drawdown. Refrigerant management.

[55] Hawkes C., Walton S., Haddad L., Fanzo J. (2020).

[56] Spiker M.L., Welling J., Hertenstein D., Mishra S., Mishra K., Hurley K.M. (2023). When increasing vegetable production may worsen food availability gaps: a simulation model in India. Food Policy.

[57] Lau W.W.Y., Shiran Y., Bailey R.M., Cook E., Stuchtey M.R., Koskella J. (2020). Evaluating scenarios toward zero plastic pollution. Science.

[58] World Economic Forum (2022). https://www.weforum.org/agenda/2022/07/india-ban-policy-single-use-plastic-pollution/.

[59] Rudel R.A., Gray J.M., Engel C.L., Rawsthorne T.W., Dodson R.E., Ackerman J.M. (2011). Food packaging and bisphenol A and bis(2-ethyhexyl) phthalate exposure: findings from a dietary intervention. Environ. Health Perspect..

[60] Hwang S., Lim J.-E., Choi Y., Jee S.H. (2018). Bisphenol A exposure and type 2 diabetes mellitus risk: a meta-analysis. BMC Endocr. Disord..

[61] Trasande L., Attina T.M., Blustein J. (2012). Association between urinary bisphenol A concentration and obesity prevalence in children and adolescents. JAMA.

[62] Ncube L.K., Ude A.U., Ogunmuyiwa E.N., Zulkifli R., Beas I.N. (2020). Environmental impact of food packaging materials: a review of contemporary development from conventional plastics to polylactic acid based materials. Materials (Basel).

[63] Crosbie E., Gomes F.S., Olvera J., Rincón-Gallardo Patiño S., Hoeper S., Carriedo A. (2022). A policy study on front-of-pack nutrition labeling in the Americas: emerging developments and outcomes. Lancet Reg. Health. Am..

[64] Potter C., Bastounis A., Hartmann-Boyce J., Stewart C., Frie K., Tudor K. (2021). The effects of environmental sustainability labels on selection, purchase, and consumption of food and drink products: a systematic review. Environ. Behav..

