# Supplementary material for: Environmental Sustainability of Food Environments: Development and Application of a Framework in 4 cities in South Asia
Source: Curr Dev Nutr. 2024 Jun 11;8(7):103791. doi: 10.1016/j.cdnut.2024.103791 (PMC11263746; doi:10.1016/j.cdnut.2024.103791)
Supplement: Multimedia component1 [file mmc1.docx]

# Supplemental Tables

**Supplemental Table 1.** Subdomains of food environments identified from previous frameworks and definitions

| **Subdomain of Food Environments** | **Framework/definition** |
| --- | --- |
| Availability | Herforth & Ahmed 2015; Turner et al 2018; Downs et al 2021; Glanz et al 2007; Glanz et al 2015; Ahmed et al 2020; Murphy et al 2017; Toure et al 2021; Sawyer et al 2021 |
| Affordability | Herforth & Ahmed 2015; Turner et al 2018; Downs et al 2021; Ahmed et al 2020; Toure et al 2021; Sawyer et al 2021 |
| Convenience | Herforth & Ahmed 2015; Turner et al 2018; Downs et al 2021; Mayer 2009; Ahmed et al 2020 |
| Desirability | Herforth & Ahmed 2015; Turner et al 2018; |
| Accessibility | Turner et al 2018; Glanz et al 2007; Glanz et al 2015; Ahmed et al 2020; Murphy et al 2017; Clary et al 2017; Toure et al 2021; Franco et al 2015 |
| Prices | Turner et al 2018; Glanz et al 2007; Glanz et al 2015; Glanz et al 2015; Global Panel 2016; Swinburn 2013; Booth et al 2021; Ahmed et al 2020; Clary et al 2017; Toure et al 2021 |
| Vendor Properties/Food Retail | Turner et al 2018; Glanz et al 2007; Glanz et al 2015; Swinburn 2013; Booth et al 2021; Cong et al 2022; Murphy et al 2017; Clary et al 2017; Toure et al 2021; Franco et al 2015 |
| Product Properties/ Food Composition | Turner et al 2018; Glanz et al 2007; Glanz et al 2015; Swinburn 2013; Booth et al 2021; Cong et al 2022 |
| Marketing and Regulation | Turner et al 2018; Ahmed et al 2020; Murphy et al 2017 |
| Promotion | Downs et al 2021; Glanz et al 2007; Glanz et al 2015; Global Panel 2016; Swinburn 2013; Booth et al 2021; Toure et al 2021 |
| Sustainable properties | Downs et al 2021; |
| Quality | Downs et al 2021; Glanz et al 2015; Global Panel 2016; Ahmed et al 2020; Toure et al 2021 |
| Safety | Global Panel 2016; Ahmed et al 2020; Toure et al 2021; |
| Food labelling | Global Panel 2016; Swinburn 2013; Booth et al 2021; |
| Food provision | Swinburn et al 2013; Booth et al 2021, |
| Food trade and investment | Swinburn et al 2013 |
| Policies | Swinburn et al 2013; Mayer 2009; Murphy et al 2017 |
| Transport | Murphy et al 2017; Toure et al 2021 |
| Land-use mix | Murphy et al 2017 |
| Acceptability | Sawyer et al 2021 |

**Supplemental Table 2.** Demographics of consumers

| **Retailer** | **Ahmedabad (n=446)** | **Pune (n=451)** | **Kathmandu (n=450)** | **Pokhara (n=450)** |
| --- | --- | --- | --- | --- |
| Age | 34.98 ± 8.08 | 41.90 ± 11.31 | 38.43 ± 12.35 | 36.47 ± 12.21 |
| Female | 98.7% (440) | 68.7% (310) | 70.4% (317) | 79.1% (356) |

Values are mean±SD or % (n)

**Supplemental Table 3.** Types of retailers surveyed in each city.

| **Retailer Type** | **Ahmedabad (n=54)** | **Kathmandu (n=55)** | **Pokhara (n=55)** | **Pune (n=66)** |
| --- | --- | --- | --- | --- |
| Food service | 0.0%  (0) | 35.4% (23) | 14.5%  (8) | 16.4%  (9) |
| Formal retailer | 90.7% (49) | 38.5% (25) | 50.9% (28) | 50.9% (28) |
| Informal retailer | 9.1%  (5) | 26.2% (17) | 34.5% (19) | 32.7% (18) |

All values are reported as % (n)

**Supplemental Table 4.** Percentage of consumers reporting purchasing food weekly by vendor type.

| **Retailer** | **Ahmedabad (n=446)** | **Pune (n=451)** | **Kathmandu (n=450)** | **Pokhara (n=450)** |
| --- | --- | --- | --- | --- |
| Government Ration Shop | 36.8% (164) | 0.7%   (3) | 2.2%  (10) | 0.4%   (2) |
| Grocery | 91.5% (408) | 17.7%  (80) | 6.7%  (30) | 0.7%   (3) |
| Internet | 7.4%  (33) | 5.8%  (26) | 0.0%   (0) | 0.0%   (0) |
| Mobile shop | 59.0% (263) | 12.4%  (56) | 43.8% (197) | 44.0% (198) |
| Permanent Wet Markets | 96.9% (432) | 33.7% (152) | 50.2% (226) | 25.8% (116) |
| Small Local Shop | 95.1% (424) | 48.8% (220) | 84.9% (382) | 90.9% (409) |
| Specialty Shop | 78.7% (351) | 41.0% (185) | 53.3% (240) | 29.8% (134) |
| Street vendor | 80.3% (358) | 27.9% (126) | 30.4% (137) | 18.7%  (84) |
| Temporary Wet Markets | 97.1% (433) | 39.7% (179) | 47.8% (215) | 23.1% (104) |

All values are reported as % (n)

**Supplemental Table 5.** Consumer purchases of food groups by food vendor type

| Food Group | Government Ration | Grocery store | Internet | Mobile vendor | Permanent Wet market | Small local shop | Specialty store | Street Vendor | Temporary Wet Market |
| --- | --- | --- | --- | --- | --- | --- | --- | --- | --- |
| Ahmedabad |  |  |  |  |  |  |  |  |  |
| Dairy | 0.2% (1) | 0.0% (0) | 0.0% (0) | 0.0% (0) | 40.0% (167) | 13.6% (57) | 43.5% (182) | 0.0% (0) | 2.6% (11) |
| Eggs | 0.0% (0) | 0.0% (0) | 0.0% (0) | 0.0% (0) | 57.1% (4) | 28.6% (2) | 0.0% (0) | 0.0% (0) | 14.3% (1) |
| Fruit and Vegetable | 0.5% (2) | 0.0% (0) | 0.0% (0) | 0.0% (0) | 45.4% (189) | 24.0% (100) | 0.0% (0) | 1.9% (8) | 28.1% (117) |
| Grain | 0.0% (0) | 1.8% (8) | 0.0% (0) | 0.0% (0) | 46.6% (207) | 24.1% (107) | 0.0% (0) | 0.0% (0) | 27.5% (122) |
| Pulses | 0.0% (0) | 3.5% (15) | 0.0% (0) | 0.0% (0) | 59.5% (256) | 19.8% (85) | 0.2% (1) | 0.0% (0) | 17.0% (73) |
| Pune | | | | | | | | | |
| Dairy | 0.0% (0) | 7.3% (27) | 1.3% (5) | 7.3% (27) | 7.5% (28) | 27.2% (101) | 45.8% (170) | 0.0% (0) | 3.5% (13) |
| Eggs | 0.3% (1) | 13.1% (40) | 1.6% (5) | 0.7% (2) | 11.8% (36) | 62.6% (191) | 5.6% (17) | 0.0% (0) | 4.3% (13) |
| Fruit and Vegetable | 0.0% (0) | 5.4% (22) | 1.2% (5) | 1.7% (7) | 31.9% (130) | 22.8% (93) | 0.0% (0) | 15.2% (62) | 21.8% (89) |
| Grain | 9.0% (36) | 47.8% (192) | 0.5% (2) | 0.5% (2) | 17.7% (71) | 18.4% (74) | 0.2% (1) | 0.0% (0) | 6.0% (24) |
| Pulses | 3.7% (15) | 49.6% (201) | 1.2% (5) | 0.5% (2) | 17.0% (69) | 21.7% (88) | 0.7% (3) | 0.0% (0) | 5.4% (22) |
| Kathmandu | | | | | | | | | |
| Dairy | 0.2% (1) | 0.0% (0) | 0.0% (0) | 3.4% (14) | 0.0% (0) | 37.0% (154) | 59.4% (247) | 0.0% (0) | 0.0% (0) |
| Eggs | 1.2% (5) | 3.7% (16) | 0.0% (0) | 0.5% (2) | 0.0% (0) | 94.6% (405) | 0.0% (0) | 0.0% (0) | 0.0% (0) |
| Fruit and Vegetable | 0.7% (3) | 0.0% (0) | 0.0% (0) | 9.1% (41) | 32.4% (146) | 37.3% (168) | 0.0% (0) | 2.2% (10) | 18.2% (82) |
| Grain | 11.3% (50) | 2.5% (11) | 0.0% (0) | 0.0% (0) | 0.2% (1) | 85.9% (379) | 0.0% (0) | 0.0% (0) | 0.0% (0) |
| Pulses | 7.8% (35) | 2.7% (12) | 0.0% (0) | 0.0% (0) | 0.2% (1) | 89.3% (401) | 0.0% (0) | 0.0% (0) | 0.0% (0) |
| Pokhara | | | | | | | | | |
| Dairy | 0.0% (0) | 0.0% (0) | 0.0% (0) | 10.0% (37) | 0.0% (0) | 45.3% (168) | 44.7% (166) | 0.0% (0) | 0.0% (0) |
| Eggs | 1.2% (5) | 0.0% (0) | 0.0% (0) | 0.7% (3) | 0.2% (1) | 97.8% (394) | 0.0% (0) | 0.0% (0) | 0.0% (0) |
| Fruit and Vegetable | 0.4% (2) | 0.0% (0) | 0.0% (0) | 8.0% (36) | 15.4% (69) | 51.4% (231) | 0.0% (0) | 1.1% (5) | 23.6% (106) |
| Grain | 7.6% (32) | 0.0% (0) | 0.0% (0) | 0.0% (0) | 0.0% (0) | 92.0% (389) | 0.0% (0) | 0.0% (0) | 0.5% (2) |
| Pulses | 7.0% (31) | 0.2% (1) | 0.0% (0) | 0.2% (1) | 0.2% (1) | 92.2% (411) | 0.0% (0) | 0.0% (0) | 0.2% (1) |

All values are reported as % (n)

**Supplemental Table 6.** Consumer mode of travel by food retailer type.

| Retailer | Transport | Ahmedabad | Pune | Kathmandu | Pokhara |
| --- | --- | --- | --- | --- | --- |
| Government Rations | Walking | 40.5% (177) | 34.0% (87) | 90.0% (233) | 87.8% (137) |
|  | Bicycle | 0.0% (0) | 0.8% (2) | 1.9% (5) | 0.6% (1) |
|  | Motorbike | 50.6% (221) | 48.8% (125) | 6.9% (18) | 8.3% (13) |
|  | Car | 0.2% (1) | 11.3% (29) | 0.0% (0) | 0.0% (0) |
|  | Public Transport | 6.6% (29) | 3.9% (10) | 0.4% (1) | 3.2% (5) |
|  | Other | 2.1% (9) | 1.2% (3) | 0.8% (2) | 0.0% (0) |
| Grocery store | Walking | 66.4% (295) | 41.4% (157) | 77.9% (155) | 55.6% (60) |
|  | Bicycle | 0.9% (4) | 0.3% (1) | 0.5% (1) | 0.0% (0) |
|  | Motorbike | 30.2% (134) | 42.0% (159) | 15.6% (31) | 25.9% (28) |
|  | Car | 0.0% (0) | 12.4% (47) | 1.0% (2) | 1.9% (2) |
|  | Public Transport | 1.1% (5) | 2.4% (9) | 5.0% (10) | 16.7% (18) |
|  | Other | 1.4% (6) | 1.6% (6) | 0.0% (0) | 0.0% (0) |
| Permanent Wet Markets | Walking | 40.5% (177) | 34.0% (87) | 90.0% (233) | 87.8% (137) |
|  | Bicycle | 0.0% (0) | 0.8% (2) | 1.9% (5) | 0.6% (1) |
|  | Motorbike | 50.6% (221) | 48.8% (125) | 6.9% (18) | 8.3% (13) |
|  | Car | 0.2% (1) | 11.3% (29) | 0.0% (0) | 0.0% (0) |
|  | Public Transport | 6.6% (29) | 3.9% (10) | 0.4% (1) | 3.2% (5) |
|  | Other | 2.1% (9) | 1.2% (3) | 0.8% (2) | 0.0% (0) |
| Small Local Shop | Walking | 74.7% (325) | 76.3% (284) | 98.7% (444) | 98.7% (441) |
|  | Bicycle | 0.2% (1) | 0.3% (1) | 0.2% (1) | 0.4% (2) |
|  | Motorbike | 22.8% (99) | 21.0% (78) | 1.1% (5) | 0.9% (4) |
|  | Car | 0.0% (0) | 1.6% (6) | 0.0% (0) | 0.0% (0) |
|  | Public Transport | 0.7% (3) | 0.5% (2) | 0.0% (0) | 0.0% (0) |
|  | Other | 1.6% (7) | 0.3% (1) | 0.0% (0) | 0.0% (0) |
| Specialty shop | Walking | 70.6% (264) | 64.9% (224) | 96.1% (366) | 87.9% (261) |
|  | Bicycle | 0.5% (2) | 0.6% (2) | 0.8% (3) | 0.3% (1) |
|  | Motorbike | 27.5% (103) | 28.1% (97) | 3.1% (12) | 7.7% (23) |
|  | Car | 0.0% (0) | 4.6% (16) | 0.0% (0) | 0.3% (1) |
|  | Public Transport | 0.3% (1) | 0.9% (3) | 0.0% (0) | 3.7% (11) |
|  | Other | 1.1% (4) | 0.9% (3) | 0.0% (0) | 0.0% (0) |
| Street Vendor | Walking | 61.6% (228) | 78.6% (173) | 91.6% (239) | 89.4% (193) |
|  | Bicycle | 0.8% (3) | 0.5% (1) | 1.5% (4) | 0.5% (1) |
|  | Motorbike | 36.2% (134) | 16.8% (37) | 6.9% (18) | 5.6% (12) |
|  | Car | 0.0% (0) | 3.2% (7) | 0.0% (0) | 0.0% (0) |
|  | Public Transport | 0.3% (1) | 0.9% (2) | 0.0% (0) | 4.6% (10) |
|  | Other | 1.1% (4) | 0.0% (0) | 0.0% (0) | 0.0% (0) |
| Temporary Wet Markets | Walking | 57.5% (252) | 62.5% (173) | 92.7% (229) | 94.1% (144) |
|  | Bicycle | 0.2% (1) | 1.1% (3) | 2.0% (5) | 1.3% (2) |
|  | Motorbike | 39.5% (173) | 29.6% (82) | 5.3% (13) | 3.3% (5) |
|  | Car | 0.0% (0) | 3.6% (10) | 0.0% (0) | 0.0% (0) |
|  | Public Transport | 1.4% (6) | 1.4% (4) | 0.0% (0) | 0.7% (1) |
|  | Other | 1.4% (6) | 1.8% (5) | 0.0% (0) | 0.7% (1) |

All values are reported as % (n)

**Supplemental Table 7.** Percentage of consumers reporting purchasing food monthly by vendor type.

| **Retailer** | **Ahmedabad (n=446)** | **Pune (n=451)** | **Kathmandu (n=450)** | **Pokhara (n=450)** |
| --- | --- | --- | --- | --- |
| Government Ration Shop | 67.3% (300) | 19.3% (87) | 20.2% (91) | 8.2% (37) |
| Grocery | 99.6% (444) | 84.0% (379) | 44.2% (199) | 24.0% (108) |
| Internet | 38.6% (172) | 31.3% (141) | 2.4% (11) | 0.2% (1) |
| Mobile shop | 73.3% (327) | 19.5% (88) | 64.2% (289) | 68.9% (310) |
| Permanent Wet Markets | 98.0% (437) | 56.8% (256) | 57.6% (259) | 34.7% (156) |
| Small Local Shop | 97.5% (435) | 82.5% (372) | 100.0% (450) | 99.3% (447) |
| Specialty Shop | 83.9% (374) | 76.5% (345) | 84.7% (381) | 66.0% (297) |
| Street vendor | 83.0% (370) | 48.8% (220) | 58.0% (261) | 48.0% (216) |
| Temporary Wet Markets | 98.2% (438) | 61.4% (277) | 54.9% (247) | 34.0% (153) |

All values are reported as % (n)
