# Supplementary material for: Environmental Sustainability of Food Environments: Development and Application of a Framework in 4 cities in South Asia
Source: Curr Dev Nutr. 2024 Jun 11;8(7):103791. doi: 10.1016/j.cdnut.2024.103791 (PMC11263746; doi:10.1016/j.cdnut.2024.103791)
Supplement: Multimedia component2 [file mmc2.docx]

# Key Informant Interview Guide

1. Our aim is to build on existing frameworks of the food environment and develop metrics for measuring sustainability and convenience. We would like to start with your initial thoughts on each framework?
   1. Probing questions: Do you think these additions to frameworks improves our understanding of food environments? Have we captured all interactions between different domains of the food environment?
2. Is there anything you would suggest we add to the framework to be more inclusive and comprehensive?
   1. Probing questions: Are all components of sustainability and/or convenience covered by the framework? Is it inclusive of different contexts including LMICs
3. How can these frameworks be more inclusive of the digital food environment given its widespread use and dependence?
   1. Probing questions: are there other components we add to sustainability and/or convenience domains to better capture digital food environments?
4. The sustainability domain has 3 main components: consumer transport, product properties, and vendor properties. Product and vendor properties have been in other food environment frameworks, but consumer transport would be new. Are there any other aspects of the built environment that should be included in the sustainability domain?

## Food Environment Metrics

1. In addition to frameworks, we have provided a list of indicators to measure each component within sustainability and/or convenience. Given your expertise in this area, are there any indicators we are missing?
2. Do you think all the indicators included measure their assigned components?
   1. Probing questions: Are there any major limitations to these indicators?
3. Since there is no “gold standard” to validate the metrics, do you have suggestions/guidance on how we should develop and test them?
4. For Sustainability, our goal is to create a metric from the listed indicators that adequately captures how environmentally sustainable a food environment is. At the moment, we are thinking of keeping sustainability metric as three subscores: a score for each component (consumer transport, vendor properties, and product properties). Do you agree with this approach?
   1. Probing questions: what are your anticipated limitations to this approach? Strengths? Any further recommendations?
